# Are Mouthwashes Really Effective against *Candida* spp.?

**DOI:** 10.3390/jof10080528

**Published:** 2024-07-29

**Authors:** Marie Maziere, Paulo Rompante, José Carlos Andrade, Célia F. Rodrigues

**Affiliations:** 1 UNIPRO—Oral Pathology and Rehabilitation Research Unit, University Institute of Health Sciences (IUCS-CESPU), 4585-116 Gandra, Portugal; marie.maziere@iucs.cespu.pt; 2 Associate Laboratory i4HB—Institute for Health and Bioeconomy, University Institute of Health Sciences—CESPU, Avenida Central de Gandra 1317, 4585-116 Gandra, Portugal; jose.andrade@iucs.cespu.pt; 3 UCIBIO—Applied Molecular Biosciences Unit, Translational Toxicology Research Laboratory, University Institute of Health Sciences (1H-TOXRUN, IUCS-CESPU), 4585-116 Porto, Portugal; 4 LEPABE—Laboratory for Process Engineering, Environment, Biotechnology and Energy, University of Porto, 4200-465 Porto, Portugal

**Keywords:** antifungal agents, oral candidiasis, mouthwash, chlorhexidine digluconate, cetylpyridinium chloride, biomedical and dental materials

## Abstract

Oral candidiasis is an opportunistic infection caused by fungi of the genus *Candida*. Nystatin, fluconazole, and miconazole are the most widely used antifungal drugs in dentistry, but in recent years, they have been shown to be less effective due to the increase in the resistance to antifungal drugs. The growing challenge of antifungal resistance emphasizes the importance of exploring not only alternative strategies in the fight against *Candida* spp. infections but also supportive treatment for pharmacological treatment for oral candidiasis. This review aims to evaluate and compare the in vitro reports on antifungal efficacy against *Candida* spp. exhibited by mouthwashes distributed on the European market. The research question was elaborated through the PEO framework recommended by PRISMA 2020. A bibliographic search strategy was developed for the scientific online databases Pubmed and ScienceDirect. According to the eligibility criteria, 21 papers were included in this study over a 27-year period. Mouthwashes containing chlorhexidine digluconate, cetylpyridinium chloride, hexetidine, and fluorine compounds among others, and natural antimicrobials, such as menthol, thymol, eucalyptol, and *Glycyrrhiza glabra* extracts, have demonstrated antifungal effectiveness. Nonetheless, the methodological variance introduces ambiguity concerning the comparative efficacy of distinct molecules or mouthwash formulations and complicates the evaluation and the comparison of results between studies. Some mouthwashes commercially available in Europe have the potential to be used in anti-*Candida* therapy and prevention since they have shown antifungal effect.

## 1. Introduction

Oral care products are used daily by billions of people all around the world [1]. The market is extensively supplied with various types of mouthwashes, also called mouth rinse, with very different purposes. Since these products are classified as “cosmetic products” on the European market, their formulation, composition, and indications focussed on marketing messages that are subject to modest regulation and little, if any, scrutiny.

Among the products used “to treat” different “diseases” of the gums and oral mucous without a medical diagnosis, without a medical prescription, and without the benefit/risk of their use having been analysed are mouthwashes that can contain synthetic and/or natural antimicrobials, such as chlorhexidine digluconate (CHX), cetylpyridinium chloride (CPC), hexetidine (HX), fluoride compounds, menthol, thymol, eucalyptol, *Glycyrrhiza glabra* extracts, and *Mentha piperita* extracts [2,3]. CHX, developed in the early 1950s (UK), is a bisbiguanide compound used as a topical broad-spectrum antimicrobial in dental practice for the treatment of inflammatory oral conditions caused by Gram-positive and Gram-negative bacteria, yeasts, and viruses. Its antimicrobial activity is dose-dependent: at lower concentrations (0.02–0.06%), CHX is bacteriostatic, and at higher concentrations (≥0.12%), it is bactericidal [4]. CPC, developed in the 1930s (USA), is a cationic quaternary ammonium compound used as a topical antiseptic in some types of mouthwashes, toothpastes, and nasal sprays in varying concentrations (0.045–0.1%) [5]. CPC has broad-spectrum antimicrobial properties (viruses, fungus, and bacteria), which act through the disorganisation in its structure and the leakage of low-molecular components out of the cell [6]. HX is a cationic antiseptic with a wide spectrum of actions against Gram-positive and Gram-negative bacteria, as well as some fungi and parasites. It is used as a 0.1% mouthwash for local infections and oral hygiene [7]. Fluorine compounds found in mouthwashes consist mainly of sodium fluoride, but we can also find calcium fluoride, potassium fluoride, stannous fluoride, and monofluorophosphate among others [8]. Fluorine compounds are usually used in mouthwashes for their remineralizing effect, but they also show antibacterial effects through their interference with the uptake and degradation of polysaccharides by the bacterial cells, and also by reducing their ability to maintain pH homeostasis [9]. Oral candidiasis is an opportunistic infection caused by fungi of the genus *Candida* associated with poor oral hygiene with the use of a removable dental prosthesis, sexual practices, and is more prevalent in immunocompromised patients [10,11,12]. About 90% of *Candida* infections are caused by five species: *Candida albicans*, *Nakaseomyces glabrataa* (formerly classified as *Candida glabrata*), *Candida tropicalis*, *Candida parapsilosis*, and *Pichia kudriavzevii* (formerly classified as *Candida krusei*) [13]. Nystatin, fluconazole, and miconazole are the most widely used antifungal drugs in dentistry, but in recent years, they have been shown to be less effective [14].

Resistance to antifungal drugs is acquired by a learning process by microorganisms, which adapt through mutations after having been exposed to a drug, making it much more difficult to eliminate [15]. The World Health Organization published a recent list of priority pathogenic fungi to focus and drive research and political interventions to strengthen the global response to fungal infections and antifungal resistance in which *C. albicans*, *C. glabrata*, *C. tropicalis*, and *C. parapsilosis* are mentioned [16,17].

The growing challenge of antifungal resistance emphasises the importance of exploring alternative strategies in the fight against *Candida* spp. infections. The aim of this study was to carry out a systematic review to evaluate and compare the antifungal efficacy against *Candida* spp. mouthwashes available on the European Market.

## 2. Methods

This study question was created in accordance with Preferred Reporting Items for Systematic Reviews and Meta-Analyses (PRISMA 2020) that strongly recommends constructing the research question in accordance with the Population, Exposure, Outcome (PEO) policy format [18]. The resulting question was as follows: which mouthwashes available in Europe have an antifungal effect against *Candida* spp.?

The study variables were defined, namely, the Minimum Inhibitory Concentration (MIC), the Minimum Fungicidal Concentration (MFC), and the Minimum Biofilm Eradication Concentration (MBEC) of mouthwashes based on CHX and/or CPC against *Candida* spp., type of assay, methodology followed, media, and time points. Variables were compiled in Microsoft Excel version 16.0.15330.20196 (32-bit) spreadsheet software (Microsoft Corporation, Redmond, WA, USA).

A specific bibliographic search strategy was developed for the PubMed and Science Direct databases up to 30 April 2024. No time limit was applied. An advanced search strategy was carried out based on keywords and MeSH (Medical Subject Headings) terms mouthwashes, antifungal agents, and *Candida* using Boolean operators. The advance search expression used was: “Mouthwashes” [Mesh] AND “Antifungal Agents” [Mesh] AND “*Candida*” [Mesh]. The revised nomenclature published in 2021 by Borman et al. was considered [13].

Inclusion criteria included in vitro and in vivo studies that cumulatively evaluated the efficacy against *Candida* spp. in isolates from the oral cavity of patients and/or in standards from the oral cavity, studies with a complete description of the materials and methods, and MIC and MFC results against *Candida* spp. Exclusion criteria included studies that evaluated *Candida* spp. in isolates not belonging to the oral cavity, mouthwashes not sold on the European market, review studies, books, and book chapters.

The risk of bias was carefully assessed to evaluate the quality of in vitro studies. Our review encompassed in vitro studies, and to assess the risk of bias, we evaluated key aspects such as the type of in vitro methodologies and completeness of outcome data. Furthermore, the risk of bias assessment was conducted primarily at the study level, considering overall quality (e.g., use of guidelines as a base to drug testing assays) and internal validity of each included study (cause-and-effect relationship). Assessment results informed the interpretation and synthesis of study findings, particularly in discussing the strength of evidence and potential limitations affecting study outcomes. Two independent reviewers were involved in the quality assessment process to minimize bias/errors. Any discrepancies in reviewers’ judgments were resolved through discussion and consensus. When consensus was not reached, a third reviewer arbitrated to achieve agreement [19].

## 3. Results and Discussion

### 3.1. Bibliographic Research

**Figure 1:**
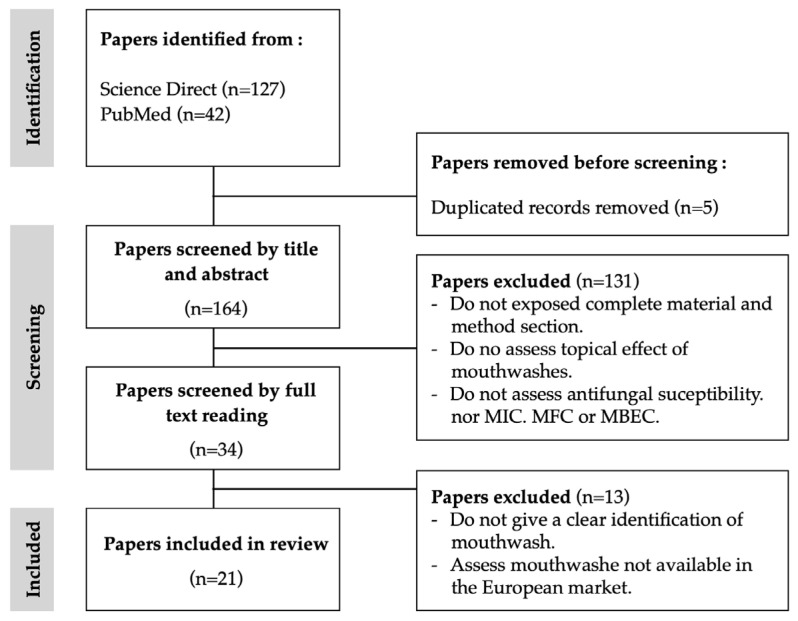
Flowchart for the bibliographic research.

After removing duplicates, the search flow diagram selected a total of 169 potentially eligible studies (Figure 1). According to the eligibility criteria, 21 papers were selected to include in this study. The time frame was 27 years, from 1997 to 2024. Notably, the period spanning from 2011 to 2020 accounted for the highest publication frequency, constituting more than 50% of the total publications. According to the inclusion criteria, only studies on mouthwash products available on the European market were included. The majority of the studies included and analysed were performed in European countries, such as Italy [2,20,21,22,23], Poland [24,25,26], Sweden-Portugal [27], Norway [27], France [28] and Finland [29,30]. The remaining ones were performed by non-European countries, namely, Chile [31], China [32], India [33], Iran [34], Malaysia [35], South Africa [36], Turkey [37], and the USA [38]. The cumulative results, as per the eligibility criteria, indicated that only two studies were in vivo studies [29,36], while the remaining encompassed in vitro experimental protocols.

### 3.2. Antifungal Activity of Active Ingredients in Mouthwashes

In the literature, there is a substantial number of mouthwashes which have been tested against *Candida* strains. In our analysis, mouthwashes were grouped according to the active substances, synthetic or natural compounds. From the analysis, it was found that most pharmaceutical laboratories or manufacturers accurately described the composition of mouthwashes; however, the opposite was also found. From the analysis, it was also noticeable that changes and mergers of pharmaceutical laboratories over time had an impact on the composition of products that maintained the same commercial identity, such as Corsodyl [27] (Table 1).

#### 3.2.1. Synthetic Active Compounds

**Figure 2:**
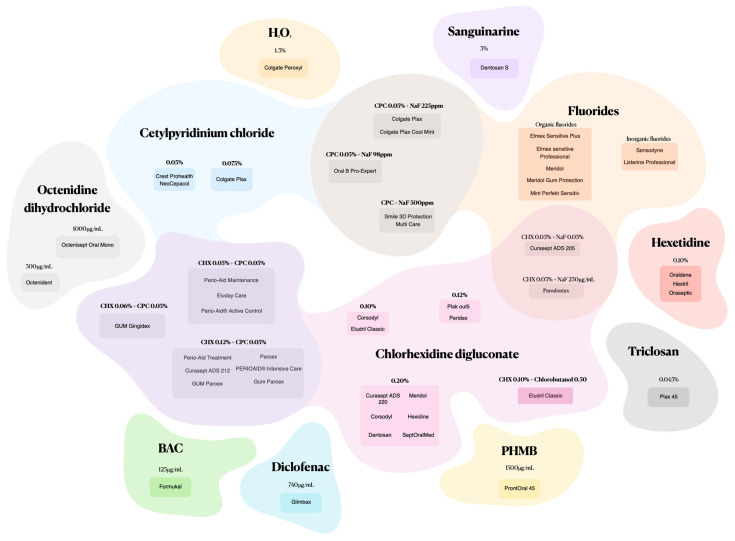
Mouthwashes composed of synthetic active ingredients and combinations.

Mouthwashes formulated with synthetic active ingredients and various combinations offer a targeted approach to candidiasis fight (Figure 2).

##### Chlorhexidine Digluconate (CHX)

**Figure 3:**
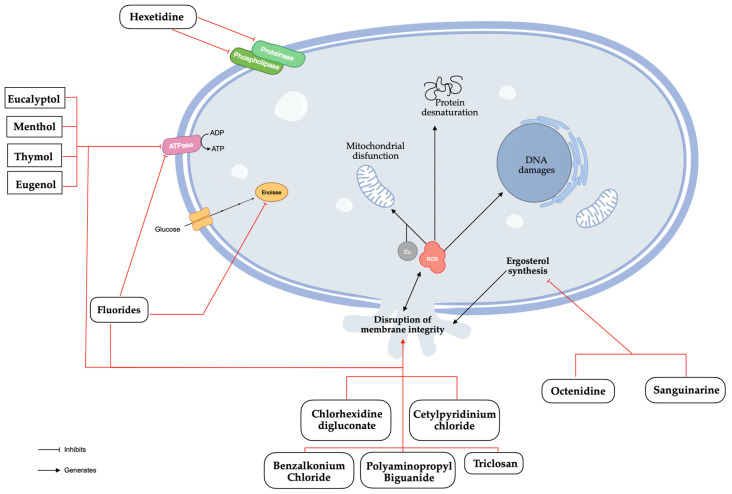
Antifungal mechanisms of natural and synthetic substances found in mouthwashes.

CHX is probably the most common antimicrobial compound present in mouthwashes with therapeutic aim in the literature [8,38]. As a positively charged di-cationic compound, CHX initially interacts with the negatively charged cell wall, subsequently binding to phospholipids in the cell membrane, leading to a leakage of potassium ions and progressive irreversible damage to the membrane and cytoplasm, ultimately resulting in bactericidal effects [41,42]. Novel studies found that CHX could induce apoptosis of *C. albicans* cells through the disruption of metal ion and ROS homeostasis, which may help to identify new targets for fungal infections (Figure 3) [43]. Potential adverse effects include tooth staining, taste changes, mouth irritation, allergic reactions, and rarely, severe irritation or chemical burns in children [6]. From our analysis, it can be stated that the concentrations of CHX found in mouthwashes were in the range of 0.1% to 0.2%, and that in the same range, the results showed similar fungistatic and fungicidal effects. *C. albicans* species showed better MIC results than non-*C. albicans Candida* species, particularly for CK and CP [24,40]. However, the result for CG was substantially better than for CA (Table 2) [24].

##### Cetylpyridinium Chloride (CPC)

CPC, a cationic quaternary ammonium compound, operates similarly to CHX by disrupting cell membrane [44]. Its antibacterial potential is well described by a disruption of the fungal membrane integrity causing a leakage of bacterial cytoplasmic components, interference with cellular metabolism, and the inhibition of cell growth [45]. The potential adverse effects include tooth staining, taste changes, gastric upset, allergic reactions, and mouth/tongue irritation [46]. CA obtained weaker MFC results than non-*C. albicans Candida* species, namely, CP and CK [21], results that were contradicted in more recent studies whose MIC, MFC, and disc diffusion assay results were worse in CA than in non-albicans species, particularly CK (Table 3) [32].

##### Hexetidine (HX)

HX belongs to the group of pyrimidine derivatives, is only found at a 0.1% concentration, and acts as an inhibitor of the phospholipase and proteinase production of *C. albicans* [37]. However, oral retention appears to be limited so that the antimicrobial activity does not last long [47]. CA showed better results in MFC compared to non-*C. albicans Candida* species in clinical isolates [21]. However, recent studies indicate that HX achieves similar inhibition percentages in CA, CG, and CK and performs better than in CP and CT in ATCC species (Table 4) [28].

##### Fluoride Compounds

While fluoride is known to inhibit bacterial metabolism, its exact mechanism in combating *Candida* species remains to be fully elucidated. Research suggests potential effects on enzyme activity and cell wall integrity. Bearing in mind that fluoride is an extremely small chemical entity and considering that when it is present in high concentrations inside cells, it displays fungicidal activity, it has led the international scientific community to test synergy models. This is the case of the association of different compounds with fungicidal activity based on mechanisms of action that disrupts the integrity of the cell membrane and of the cell [46,48]. The results between CA and non-*C. albicans Candida* species are inconsistent. In the disc diffusion assay, the inhibition halos for the ELMEX mouthwash were smaller for CA than for CG, CK, and CP standard species. However, the Mint Perfect Sensitive mouthwash showed better results in CA compared to non-*Candida albicans Candida* species (Table 5) [26].

##### Combinations of Substances

These developments explain the emergence of the combination of active substances in mouthwashes, such as the combination of CHX and CPC, or CHX and fluoride, a strategy that has been shown to increase fungicidal activity and reduce toxicity [26]. Results highlighted that the association of CHX with CPC were moderately dependent on the concentration of CHX; in fact, the antifungal effect between CHX 0.05% + CPC 0.05% and CHX 0.12% + CPC 0.05% were very similar [31]. According to the literature, CHX is often associated with CPC to improve antifungal results [44] but in the papers studied, CHX 0.10% undoubtedly obtained better results than CHX 0.12% + CPC 0.05% due to excipients [25].

The antifungal effect of the combination of CPC and NaF is more effective on non-C. *albicans Candida* standard species than on CA regardless of the NaF concentration (Table 6) [26,32].

##### Other Substances

Octenidine (OCT), sanguinarine (SNG), benzalkonium chloride (BAC), polyaminopropyl biguanide (PHMB), and triclosan (TCS) are occasionally found in other mouthwashes. OCT and SNG have inhibitory effects on ergosterol’s biosynthesis which lead to the generation of ROS and the disruption of the membrane integrity [49,50]. BAC is known to denature proteins and disrupt the membrane integrity through penetration of the hydrophobic bilayer, compromising cellular permeability controls and causing cell leakage and lysis [51]. PHMB interacts with the cell membrane through electrostatic interactions and disrupt it, followed by an accumulation within the cytosol where it disrupts the nuclear membrane and fragments DNA [52]. TCS disorganizes the microbial cytoplasmic membrane inducing microorganisms’ lysis [53]. In January 2009, the Scientific Committee on Consumer Safety (SCCS) adopted an opinion on its human health safety, considering that TCS should only be used as a preservative in mouthwashes at a maximum concentration of 0.2% [54]. Given the current data, it is important to consider that the observed results may be attributed, at least in part, to the presence of TCS. In clinical isolates, TCS has a similar effect on CA, CK, and CP, but MFC is worse for CK and CP (Table 7) [21].

#### 3.2.2. Natural Active Compounds

Menthol, eucalyptol, thymol, and eugenol are the main natural active ingredients found in mouthwashes. Menthol, thymol, eugenol, and eucalyptol have similar mechanisms of action. They disrupt biosynthetic and signalling pathways, causing mitochondrial dysfunction by inhibiting the electron transport system, reducing mitochondrial transmembrane potential, blocking respiratory proton pumps, decreasing ATP production, and increasing ROS generation, which ultimately triggers apoptosis [55,56,57,58]. The MFC results were similar across all species, but the MIC results were better for CA, CP, and CD compared to CG, CK, and CT [40]. However, recent studies have not confirmed these findings for *C. albicans* species, which show lower MIC, MFC, and disc diffusion results (Table 8) [29,32,33]. 

#### 3.2.3. Excipients

Regrettably, the literature does not directly address the potential role of excipients in the antifungal activity of mouthwashes. However, excipients can influence the bioavailability of active antifungal compounds by enhancing absorption or altering release kinetics. This can lead to higher concentrations of active ingredients at the target site, boosting antifungal efficacy. Consequently, mouthwashes with the same active ingredients but different excipients may exhibit varying antimicrobial effectiveness.

Nonetheless, some excipients used in mouthwashes, such as preservatives, surfactants, or solvents may have inherent antimicrobial properties that could contribute to the overall antifungal activity of the formulation. For instance, sodium benzoate, methylparaben, and ethylparaben prevent microbial growth in mouthwashes [8,59]. Others, such as ethanol, 2-propanol, dichlorobenzyl alcohol, and phenethyl alcohol have also shown antimicrobial efficacy [60,61].

Potential adverse effects of the chronical use of alcohol-based mouthwashes may further increase the risk of developing an oral cancer where other risk factors for oral cancer are present [62].

### 3.3. Bias and Studies’ Limits

Most authors assume that mouthwashes only have one active substance in their composition, and that this substance is the only one that may or may not have fungicidal activity. Studies that have tested the antifungal effect of mouthwashes against microorganisms in the biofilm phase are scarce, as most focus on planktonic phases [25,29] However, it is acknowledged that *Candida* spp. can form biofilms to increase its survival by secreting an extracellular matrix, increasing its adhesion to oral mucous membranes, and to the gingiva in particular, increasing colonization through its ability to create hyphae, pseudohyphae, and polyspecies biofilms, as confirmed in several reports [63,64]. Thus, the published results regarding the effectiveness of mouthwashes against *Candida* spp. may be overestimated, since the methodological scenarios do not include colonization in the biofilm phase, where the resistance is clearly greater to antifungal agents, which in turn is a treat to global health. Naturally, this highlights the importance of the biofilm phase in the research plans for the development of antifungal therapies for oral infections, as stated in the literature [65,66]. 

The risk of bias in a study is the key point for many researchers; however, the importance and analysis of the transposition of in vitro models to in vivo models cannot be neglected. Hence, we risk saying that the analysis in many cases must undergo additional scrutiny and verify whether models are realistic or not. From our study, it is clear that there is a great diversity of methods, especially of co-culturing time points. The method that most closely mimics in vivo conditions under which mouthwashes are administered may be considered the most realistic: rinsing for 30–60 s (time recommended by most manufacturers) [2,20,22,23,29,32,36]. Nevertheless, the substantivity of antiseptics in saliva is around 6 h and reaches 11 h in the oral dental film, the interdental zone, the anterior labial mucosa, and the posterior buccal mucosa [67]. Therefore, more articles become relevant because they test longer time scales [23,35,39,40]. More than analysing the potential bias of a study, the diversity of methods poses great difficulty when comparing results and in the consistency of the analysis of the results, which in itself can even be an interpretation bias when comparing results (Table 9).

Thus, the lack of standardized methodologies (gold standard protocols) is the first major difficulty for comparing results. It complicates the evaluation, the comparison of results from diverse studies, and does not allow scientific rigor when stating which mouthwash has the best antifungal effect. On the other hand, the real in vivo conditions in which oral mouthwashes are used are very far from the conditions in which oral mouthwashes are tested, to the point that the tested study models may be considered, within the context of a constructive scientific analysis, unrealistic. This is clear for no other reason than the contradiction among manufacturers’ recommendations regarding the contact time of mouthwashes [2,20,22,23,29,32,36] and the contact time of the different study models tested. Even when considering the substantivity of oral mouthwashes in saliva, this contradiction is not solvable and calls for more accurate models, as has already been raised in the literature [23,35,39,40]. Once there, with standardized and realistic methods, it will be necessary to invest in a bias analysis, which at this moment seems to us to be of little relevance from a scientific point of view.

## 4. Conclusions

While conducting this review, significant methodological variabilities across the studies included in the work were revealed. Several key factors contributing to this issue, such as differences in study design, variation in *Candida* strains, and the use of different methodologies were identified. This variability presents a challenge in drawing definitive conclusions at an absolute level, as it can result in a significant impact on the outcomes and limit the comparability of the results. For example, different strains may respond differently to the same mouthwash, making it difficult to generalize the findings across all studies. Also, the methodologies used to measure the anti-*Candida* spp.’s effectiveness varied, including disparities in the type of assays, endpoints, and criteria for determining success, which, per se, can lead to different interpretations of the effectiveness of the mouthwashes. Also, given these methodological differences, this study focused on identifying trends and general observations rather than attempting to establish definitive rankings or absolute conclusions. Indeed, with the available data, it is not possible to state that the oral mouthwashes available on the European market, regardless of their composition, are generally effective against *Candida* spp. It can only be admitted that oral mouthwashes made from CHX, CPC, HX, fluorinated compounds, and natural compounds demonstrated antifungal efficacy in in vitro study models; critically, no consideration was given to the excipients that are part of the pharmaceutical preparation.

We also underscore the importance of developing standardized protocols for future research to enable more accurate and reliable comparisons. Until such standardization is achieved, our conclusions remain tentative and should be interpreted within the context of the noted methodological variability. Future investigations are suggested in standardized studies using realistic in vivo models that can assess the effectiveness of oral mouthwashes in oral candidiasis associated with traditional pharmacological treatment to define more effective clinical protocols.

## Figures and Tables

**Table 1:** Mouthwashes analysed in previous studies (by alphabetic order).

Mouthwash	Manufacturer	Active Substance	Paper
Andolex	3M, Maplewood, MN, USA	Benzydamine hydrochloride + CHX	[36]
Baikadent mint	Herbapol, Lublin, Poland	*Scutellaria baicalensis* root extract	[25]
Cariax Gingival	Laboratorios Kin SA, Barcelona, Spain	CHX 0.12% + NaF 0.05%	[39]
Chlorexidine	Foramen, Guarnizo, Spain	Pro-vitamin B5 0.50% + CHX 0.12% + NaF 0.05%	[34]
Chlorhexamed-Fluid	Proctor & Gamble GmbH, Cincinnati, OH, USA	CHX 0.10%	[39]
Colgate	Colgate Palmolive, New York, NY, USA	H_2_O_2_ 1.5%	[29]
Colgate Peroxyl		-	[34]
Colgate Plax		CPC 0.05% + F^−^ 225 ppm	[32]
		CPC 0.075%	[31]
Colgate Plax Complete Care Sensitive		-	[24]
Colgate Plax Cool Mint		CPC + NaF 225 ppm	[26]
Corpore Sano	Corpore Sano, Plymouth, MI, USA	Extracts of *Foeniculum vulgare* + *Commiphora myrrha + Propolys*	[34]
Corsodyl	SmithKline Beecham, London, UK	CHX 0.20%	[21]
	GlaxoSmithKline, Brentford, UK	CHX 0.10%	[27]
		CHX 0.20%	[39]
			[29]
			[24]
			[25]
Crest Prohealth Mouthwash	Procter and Gamble, Cincinnati, OH, USA	CPC 0.05%	[32]
Curasept ADS 220	Curasept, Saronno, Italy	CHX 0.20%	[23]
			[20]
Curasept ADS 205		СНХ 0.05% + NaF 0.05%	[24]
			[2]
Curasept ADS 212		CHX 0.12% + CPC 0.05%	[2]
Dentosan	Warner Wellcome, Morris Plains, NJ, USA	CHX 0.20%	[21]
	Recordati, Milan, Italy	CHX 0.20%	[23]
			[20]
Dentosan S	Warner Wellcome, Morris Plains, NJ, USA	SNG 3%	[21]
Dentosept	Phytopharm, Kleka, Poland	-	[24]
		Extract from *Salviae folium*, *Menthae piperitae* herba, Thymi herb, *Matricariae* flos *Quercus cortex, Arnica* herba, *Calami rhizomate* + benzocaine	[25]
Elmex	Colgate Palmolive, New York, NY, USA	Olaflur + NaF 0.025%	[26]
Elmex Sensitive Plus		Olaflur + KF (250 μg/mL)	[25]
Elmex sensitive Professional		Fluorine-containing molecules	[23]
			[20]
Eluday Care	Pierre Fabre, Paris, France	CHX 0.05% + CPC 0.05%	[2]
Eludril Classic	Pierre Fabre, Paris, France	CHX 0.10% + chlorobutanol 0.50%	[2]
			[25]
Fomukal	Vipharm, Ozarow, Poland	BAC 125 μg/mL	[25]
Glimbax	Angelini Pharma, Roma, Italy	Diclofenac (740 μg/mL)	[25]
GUM Paroex	Sunstar, Etoy, Switzerland	CHX 0.06% + CPC 0.05%	[2]
		CHX 0.12% + CPC 0.05%	[2]
			[25]
Hexidine	ICPA Health Products, Mumbai, India	CHX 0.20%	[33]
Hexoral	Warner-Lambert, Morris Plains, NJ, USA	HX 0.1%	[39]
Hextril	Johnson & Johnson, New Brunswick, NJ, USA	HX 0.1%	[28]
Listerine		Eucalyptol 0.092% + thymol 0.064% + methyl salicylate 0.060% + menthol 0.042% + ethanol 26.9%	[40]
			[39]
			[29]
			[33]
			[32]
			[20]
			[31]
Listerine Professional		NaF 220 ppm	[26]
Listerine Total Care		NaF 220 μg/mL + eucalyptol + thymol + menthol + alcohol	[23]
			[25]
Meridol	Colgate Palmolive, New York, NY, USA	CHX 0.20%	[23]
			[20]
		Fluorine-containing molecules	[23]
		Olaflur + SnF2 250 μg/mL	[39]
			[30]
			[25]
Mint Perfekt Sensitiv	Ziaja, Gdansk Poland	Olaflur + NaF 0.025%	[26]
NeoCepacol	Gruppo Lepetit, Valcanello Anagni, Italy	CPC 0.05%	[21]
Octenident	Schülke & Mayr, Norderstedt, Germany	Octenidine HCl 500 μg/mL	[25]
Octenidol		-	[24]
Octenisept Colourless		Octenidine dihydrochloride 0.1% + 2-phenoxy-ethanol 2.0%	[39]
Octenisept Oral Mono		Octenidine dihydrochloride 1000 μg/mL	[25]
Odol-med 3 depot	SmithKline Beecham GmbH & Co., London, UK	CHX 0.06% + p-hydroxyalkyl benzoate 0.2%	[39]
Oral-B	Procter and Gamble, Cincinnati, OH, USA	-	[34]
Oral-B		CPC 0.05% + NaF 0.05%	[20]
Oral-B Pro-Expert		CPC 0.05% + NaF 98 ppm	[26]
Oral-B Pro-Expert Clinic Line		-	[24]
Oraldene	Warner-Lambert, Morris Plains, NJ, USA	HX 0.1%	[29]
Oraseptic	Warner Wellcome, Morris Plains, NJ, USA	HX 0.1%	[21]
Parodontax	Stafford-Miller, Waterford, Ireland	CHX 0.06% + NaF 250 ppm	[39]
	GlaxoSmithKline, Brentford, UK		[23]
			[20]
Peridex	3M, Maplewood, MN, USA	CHX 0.12%	[40]
Perio Aid Intensive Care	Dentaid, Barcelona, Spain	CHX 0.12% + CPC 0.05%	[31]
			[2]
			[25]
Perio plus	Mooss-Pharma, Izegem, Belgium	CHX 0.12% + 2-phenoxyethanol 0.05%	[39]
Perio-Aid Active Control	Dentaid, Barcelona, Spain	CHX 0.05% + CPC 0.05%	[31]
			[2]
Plak out5	Colgate Palmolive, New York, NY, USA	CHX 0.12%	[21]
PlaKKontrol Protezione Totale	IDECO, Bolzano, Italy	TCS + NaF	[20]
Plax	Colgate Palmolive, New York, NY, USA	TCS + NaF	[36]
Plax 45		TCS 0.045%	[21]
ProntOral	B Braun, Melsungen, Germany	PHMB 1500 μg/mL	[25]
Santoin	Walmark spol, Třinec, Czech Republic	Menthol + thymol + extract of *Macleaya cordata*	[39]
Sensodyne	GlaxoSmithKline, Brentford, UK	NaF	[34]
SeptOralMed	Avec Pharma, Wroclaw, Poland	CHX 0.20%	[25]
Smile 3D Protection Multi Care	Smile 3D Protection, Santa Rosa, Philippines	CPC + NaF 500 ppm	[26]
Sylveco Herbal Mouthwash	Sylveco, Łąka, Poland	*Salvia Officinalis* leaf extract, *Mentha Piperita* leaf extract, *Rosmarinus Officinalis* leaf extract, *Eugenia Caryophyllus* flower extract, *Mentha Piperita* oil	[24]
Total	Colgate Palmolive, New York, NY, USA	TCS 0.03% + sodium methylcocoyl taurate 0.25% + polyvinylmethyl ether/maleic acid copolymer 1.0%	[39]

BAC: benzalkonium chloride; CHX: chlorhexidine digluconate; CPC: cetylpyridinium chloride; HX: hexetidine; KF: potassium fluoride; NaF: sodium fluoride; PHMB: polyaminopropyl biguanide; SNG: sanguinarine; TCS: Triclosan.

**Table 2:** Antifungal effect results of chlorhexidine digluconate (CHX)-based mouthwashes.

C	Mouthwash	*Candida* spp.			Antifungal Results				Paper
		Species	CI	Reference Strain	MIC	MFC	DI (mm)	% Inhibition	
**0.10%**	Corsodyl	CA		SC5314				R	[27]
	Eludril Classic		X	ATCC 10231, ATCC 14053	0.12%	0.12%		41.7% B	[25]
**0.12%**	Plak out5	CA	X			37.5 μg/mL			[21]
		CP	X			150 μg/mL			
		CK	X			9.37 μg/mL			
	Peridex	CL	X		0.6 μg/mL	4.7 μg/mL			[40]
		CK	X	X	1.2 μg/mL	4.7 μg/mL			
		CP	X		1.2 μg/mL	9.4 μg/mL			
		CD	X	NCPF	2.3 μg/mL	6.4 μg/mL			
		CG	X		2.3 μg/mL	4.7 μg/mL			
		CT	X		2.3 μg/mL	4.7 μg/mL			
		CA	X	ATCC18804	9.4 μg/mL	9.4 μg/mL			
**0.20%**	Corsodyl	CA	X			31.25 μg/mL			[21]
			X	ATCC 90028	0.003	0.003			[29]
			X		0.1671		14.5		[24]
			X	ATCC 10231, ATCC 14053	0.12%	0.12%		32.6% B	[25]
		CG	X		0.115		16.5		[24]
			X		0.2698		13.2		[24]
		CK	X			7.81 μg/mL			[21]
			X		0.2185		19.3		[24]
		CP	X			62.25 μg/mL			[21]
			X		0.262		16.2		[24]
		CT	X		0.2719		16.6		
	Curasept ADS 220	CA		SC5314			11 ± 0		[23]
	Dentosan	CA	X			31.25 μg/mL			[21]
				SC5314			14 ± 0		[23]
		CK	X			7.81 μg/mL			
		CP	X			250 μg/mL			
	Hexidine	CA	X		0.0313				[33]
		CT	X		0.0313				
	Meridol	CA		SC5314			13 ± 0		[23]
	SeptOralMed	CA	X	ATCC 10231, ATCC 14053	0.12%	0.12%		32.1% B	[25]

B: biofilm; C: concentration; CI: clinically isolated; CA: *Candida albicans*; CK: *Candida krusei*; CG: *Candida glabrata*; CT: *Candida tropicalis*; CP: *Candida parapsilosis*; CD: *Candida dubliniensis*; CL: *Candida lusitania*; DI: diameter of inhibition.

**Table 3:** Antifungal effect results of cetylpyridinium chloride (CPC)-based mouthwashes.

C	Mouthwash	*Candida* spp.			Antifungal Results			Paper
		Species	CI	Reference Strain	MIC	MFC	DI (mm)	
0.05%	Crest Prohealth Mouthwash	CK		ATCC 6258	0.10 μg/mL	0.10 μg/mL	16 ± 1.0	[32]
		CA	X	ATCC 90028	0.53 μg/mL	0.72 μg/mL	10.8 ± 2.5	
	NeoCepacol	CA	X		-	7.81 μg/mL		[21]
		CP	X		-	1.95 μg/mL		
		CK	X		-	1.95 μg/mL		
0.075%	Colgate Plax	CA	X		-	-	20.5 ± 2.66	[31]

C: concentration; CI: clinically isolated; CA: *Candida albicans*; CK: *Candida krusei*; CP: *Candida parapsilosis*; DI: diameter of inhibition.

**Table 4:** Antifungal effect results of hexetidine (HX)-based mouthwashes.

C	Mouthwash	*Candida* spp.			Antifungal Results			Paper
		Species	CI	Reference Strain	MIC	MFC	% Inhibition	
0.10%	Oraldene	CA	X	ATCC 90028	0.012	0.0327		[29]
	Hextril	CA		ATCC 90028			100% P	[28]
		CP		ATCC 22019			81% P	
		CG		ATCC 90030			100% P	
		CK		ATCC 6258			100% P	
		CT		ATCC 1731			97% P	
	Oraseptic	CA	X		-	31.25 μg/mL		[21]
		CP	X		-	15.62 μg/mL		
		CK	X		-	7.81 μg/mL		

C: concentration; CI: clinically isolated; CA: *Candida albicans*; CK: *Candida krusei*; CG: *Candida glabrata*; CT: *Candida tropicalis*; CP: *Candida parapsilosis*; P: planktonic cell solution.

**Table 5:** Antifungal effect results of fluorine-based mouthwashes.

C	Mouthwash	*Candida* spp.			Antifungal Results				Paper
		Species	CI	Reference Strain	MIC	MFC	DI (mm)	% Inhibition	
250 μg/mL	Listerine Professional	CA		ATCC 10231			0		[26]
		CG		ATCC 90030			0		
		CP		ATCC 22019			0		
		CK		ATCC 14243			0		
	Meridol	CA		ATCC 2091, ATCC 10231	1–4				[30]
		CP		ATCC 20019	1–4				
		CG		ATCC 90030	1–4				
		CK		ATCC 14243	1–4				
	Elmex	CA		ATCC 10231			13.5		[26]
		CG		ATCC 90030			17.5		
		CP		ATCC 22019			16		
		CK		ATCC 14243			14.5		
	Elmex Sensitive Plus	CA	X	ATCC 10231, ATCC 14053	47.92%	47.92%		28.4% B	[25]
	Meridol Gum Protection		X		43.75%	43.75%		27.2% B	
	Mint Perfekt Sensitiv	CA		ATCC 10231			8		[26]
		CG		ATCC 90030			0		
		CP		ATCC 22019			0		
		CK		ATCC 14243			0		
unknown	Sensodyne	CA		PTCC5027	0.03 mg/L		19.87 ± 1.32		[34]
	Elmex sensitive Professional			SC5314			R		[23]
	Meridol						R		

B: biofilm; C: concentration; CI: clinically isolated; CA: *Candida albicans*; CG: *Candida glabrata*; CK: *Candida krusei*; CP: *Candida parapsilosis*; DI: diameter of inhibition; R: resistant.

**Table 6:** Antifungal effect results of pharmacological combinations.

Mixture		Mouthwash	*Candida* spp.			Antifungal Results				Paper
			Species	CI	Reference Strain	MIC	MFC	DI (mm)	% Inhibition	
CHX 0.05%	CPC 0.05%	Perio-Aid Maintenance	CA	X		-	-	24.6 ± 2.62		[31]
		Eluday Care		X		0.04%	0.04%			[2]
		Eluday Care			ATCC 10231	0.09%	0.09%			
		PERIOAID^®^ Active Control			ATCC 10231	0.02%	0.02%			
		PERIOAID^®^ Active Control		X		0.04%	0.04%			
	NaF 0.05%	Curasept ADS 205	CA	X		0.02%	0.09%			
					ATCC 10231	0.09%	0.09%			
				X		0.1186		14.1		[24]
			CG	X		0.1489		14.1		
			CP	X		0.1668		15		
			CT	X		0.455		13.6		
			CK	X		0.1824		15		
CHX 0.06%	CPC 0.05%	GUM Gingidex	CA	X		0.02%	0.02%			[2]
		GUM Gingidex			ATCC 10231	0.0009	0.0009			
	NaF 250 μg/mL	Parodontax			SC5314			9 ± 0		[23]
CHX 0.10%	Chlorobutanol 0.50%	Eludril Classic	CA		ATCC 10231	0.04%	1.56%			[2]
		Eludril Classic		X		0.04%	0.04%			
CHX 0.12%	CPC 0.05%	Perio-Aid Treatment	CA	*X*		-	-	25.65 ± 2.39		[31]
		Curasept ADS 212		X		0.02%	0.02%			[2]
					ATCC 10231	0.09%	0.09%			
		GUM Paroex		X		0.04%	0.04%			
		Paroex			ATCC 10231	0.04%	0.04%			
		PERIOAID^®^ Intensive Care		X		0.04%	0.04%			
					ATCC 10231	0.09%	0.09%			
				X	ATCC 10231 and 14053	0.13%	0.13%		29.2% (B)	[25]
		Gum Paroex		X	ATCC 10231 and 14053	0.13%	0.13%		27.6% (B)	
	NaF 0.05%	Chlorexidine			PTCC5027	0.062 mg/L		R		[34]
CPC 0.05%	NaF 225 ppm	Colgate Plax	CK		ATCC 6258	0.05 μg/mL	0.10 μg/mL	12.2 ± 1.1		[32]
			CA		ATCC 90028	0.58 μg/mL	0.67 μg/mL	13.4 ± 3.5		
		Colgate Plax Cool Mint	CA		ATCC 10231			19.5		[26]
			CG		ATCC 90030			21.5		
			CP		ATCC 22019			20.5		
			CK		ATCC 14243			23.5		
CPC 0.05%	NaF 98 ppm	Oral B Pro-Expert	CA		ATCC 10231			18		
			CG		ATCC 90030			20.5		
			CP		ATCC 22019			19.5		
			CK		ATCC 14243			20.5		
CPC	NaF 500 ppm	Smile 3D Protection Multi Care	CA		ATCC 10231			20.5		
			CG		ATCC 90030			20.5		
			CP		ATCC 22019			20.5		
			CK		ATCC 14243			21.5		

B: biofilm; C: concentration; CI: clinically isolated; CA: *Candida albicans*; CK: *Candida krusei*; CG: *Candida glabrata*; CT: *Candida tropicalis*; CP: *Candida parapsilosis*; DI: diameter of inhibition.

**Table 7:** Antifungal effect results of mouthwashes with other pharmacologic active substances.

Substance	Mouthwash	*Candida* spp.			Antifungal Results			Paper
		Species	CI	Reference Strain	MIC	MFC	% Inhibition	
**BAC (125 μg/mL)**	Fomukal	CA	X	ATCC 10231, ATCC 14053	6.38%	6.51%	35.7% B	[25]
**Diclofenac (740 μg/mL)**	Glimbax		X	ATCC 10231, ATCC 14053	70.83%	75.00%	26.4% B	
**H_2_O_2_ 1.5%**	Colgate Peroxyl		X	ATCC 90028	0.012	0.0327		[29]
**Octenidine dihydrochloride (1000 μg/mL)**	Octenisept Oral Mono		X	ATCC 10231, ATCC 14053	0.09%	0.09%	51.1% B	[25]
**Octenidine HCl (500 μg/mL)**	Octenident		X	ATCC 10231, ATCC 14053	0.10%	0.10%	47.0% B	
**PHMB (1500 μg/mL)**	ProntOral		X	ATCC 10231, ATCC 14053	1.89%	1.89%	38.6% B	
**SNG 3%**	Dentosan S		X		-	15,000 μg/mL		[21]
		CP	X		-	>		
		CK	X		-	15,000 μg/mL		
**TCS 0.045%**	Plax 45	CA	X		-	14.06 μg/mL		
		CP	X		-	14.06 μg/mL		
		CK	X		-	14.06 μg/mL		

B: biofilm; C: concentration; CI: clinically isolated; CA: *Candida albicans*; CK: *Candida krusei*; CP: *Candida parapsilosis*; TCS: Triclosan

**Table 8:** Antifungal effect results of mouthwashes with natural active compounds.

Composition	Mouthwash	*Candida* spp.			Antifungal Results				Paper
		Species	CI	Reference Strain	MIC	MFC	DI (mm)	% Inhibition	
*Scutellaria baicalensis* root extract	Baikadent mint	CA	X	ATCC 10231, ATCC 14053	31.25%	33.33%		35.6% B	[25]
Extracts of *Foeniculum vulgare* + *Commiphora myrrha* + Propolys	Corpore Sano	CA		PTCC5027	0.062 mg/L		R		[34]
Extract from *Salviae folium*, *Menthae piperitae herba*, *Thymi herb*, *Matricariae flos*, *Quercus cortex*, *Arnica herba*, *Calami rhizomate* + benzocaine	Dentosept	CA	X	ATCC 10231, ATCC 14053	45.83%	45.83%		-	[25]
	Dentosept A	CA	X		0.2293		17.1		[24]
		CG	X		0.1984		16.2		
		CP	X		0.1263		14.2		
		CT	X		0.1339		17.3		
		CK	X		0.25		17		
Eucalyptol (0.092%), thymol (0.064%), methyl salicylate (0.060%), menthol (0.042%), and ethanol (26.9%)	Listerine	CA		ATCC 90028	44.44 μg/mL	-	8 ± 3.4		[32]
		CK		ATCC 6258	6.25 μg/mL	12.50 μg/mL	8.2 ± 0.6		
		CA	X	ATCC 90028	25%	25%			[29]
		CT	X		6.25%	-			
		CA	X		12.50%	-			
NaF 220 μg/mL, eucalyptol, thymol, menthol, alcohol	Listerine Total Care Zero	CA		SC5314			R		[23]
	Listerine Total Care	CA	X	ATCC 10231, ATCC 14053	16.67%	18.23%		33.2% B	[25]
	Listerine Zero	CA	X		-	-	R		[31]
Thymol (0.064%), eucalyptol (0.092%), methyl salicylate (0.060%), and menthol (0.042%)	Listerine Tartar Control	CL	X		161.2 μg/mL	645 μg/mL			[40]
		CA	X	ATCC 18804	322.5 μg/mL	645 μg/mL			[40]
		CD	X	NCPF	322.5 μg/mL	645 μg/mL			
		CP	X	CI	322.5 μg/mL	645 μg/mL			
		CG	X	CI	645 μg/mL	645 μg/mL			
		CK	X	X	645 μg/mL	645 μg/mL			
		CT	X		645 μg/mL	645 μg/mL			
*Salvia Officinalis* leaf extract, *Mentha Piperita* leaf extract, *Rosmarinus Officinalis* leaf extract, *Eugenia Caryophyllus* flower extract, *Mentha Piperita oil*	Sylveco Herbal Mouthwash	CA	X		0.1345		17.1		[24]
		CG	X		0.2902		16.2		
		CP	X		0.1231		14.2		
		CT	X		0.1465		17.3		
		CK	X		0.0954		17		

B: biofilm; CI: clinically isolated; CA: *Candida albicans*; CK: *Candida krusei*; CG: *Candida glabrata*; CT: *Candida tropicalis*; CP: *Candida parapsilosis*; CD: *Candida dubliniensis*; CL: *Candida lusitania*; DI: diameter of inhibition; R: Resistant.

**Table 9:** Contact time between mouthwashes and *Candida* strains by assay type.

Paper	Assay Type					
	Broth Macrodilution	Broth Microdilution	Time-Kill Assay	Biofilm Testing	In Vivo Assays	Other
[21]	24 h	-	0–180 s (15 s interval)	-	-	-
[22]	24 h	-	-	-	-	-
[40]	24 h and 48 h	-	90 s, 120 s, 150 s, 180 s, 210 s	-	-	-
[39]	-	-	-	16.5 h, 20.5 h, 24.5 h, 40.5 h, 44.5 h, and 48.5 h	-	-
[36]	-	-	-	-	60 s (12 weeks)	-
[30]	24 h	-	-	-	-	-
[33]	24 h	-	0 min, 30 min, 60 min, 90 min, 120 min	-	-	-
[29]	-	-	-	-	30–60 s	-
[35]	24 h	-	480 min (15 min interval)	-	-	-
[28]	-	-	-	-	-	5 min
[34]	-	24 h	-	-	-	-
[32]	-	48 h	-	30, 60, and 120 s	-	-
[37]	-	30 min	-	-	-	-
[23]	-	-	-	1 min, 5 min, 10 min, 30 min	-	-
[20]	-	-	-	1 min	-	-
[27]	-	-	-	24 h	-	-
[31]	-	-	-	-	-	24 h
[24]	-	-	-	-	-	-
[26]	-	-	-	48 h	-	-
[2]	24 h	-	10 s, 30 s, 60 s, 5 min, 15 min, 30 min, and 60 min	-	-	-
[25]	-	24 h	-	24 h	-	-

## Data Availability

Not applicable.
